# Trends in clinical trials for stroke by cell therapy: data mining ClinicalTrials.gov and the ICTRP portal site

**DOI:** 10.1038/s41536-019-0082-7

**Published:** 2019-11-06

**Authors:** Takaharu Negoro, Hanayuki Okura, Midori Maehata, Shigekazu Hayashi, Satoru Yoshida, Nozomi Takada, Akifumi Matsuyama

**Affiliations:** Department of Regenerative Medicine Support Promotion Facility, Center for Research Promotion and Support, Fujita Health University, Toyoake, 470-1192 Japan

**Keywords:** Translational research, Mesenchymal stem cells

## Abstract

Definitive treatment of stroke constitutes an important thesis of regenerative medicine in the cerebrovascular field. However, to date, no cell therapy products for stroke are yet on the market. In this study, we examined the clinical research trends related to cell therapy products in the stroke field based on data obtained from the ClinicalTrials.gov website and International Clinical Trials Research Platform (ICTRP) portal site. These data do not offer results of clinical trials comprehensively but provide information regarding various attributes of planned clinical trials including work in progress. We selected 78 cell therapy studies related to the field of stroke treatment from ClinicalTrial.gov and ICTRP. These were analyzed according to, e.g., the reporting countries, origin (autologous or allogeneic), of cell used, cell types and source organs, the progress of translational phases, target phase of the disease (acute or chronic stroke), and route of administration. This analysis revealed a trend whereby in the acute phase, mesenchymal stem cells were administered intravenously at a relatively higher dose, whereas in the chronic phase a small number of cells were administered intracranially. Only two randomized controlled Phase III studies with over 100 patients are registered, but none of them has been completed. Thus, cell therapy against stroke appears to constitute a premature area compared with cartilage repair as assessed in our previous report. In addition, tracking by means of the ID number of each trial via PubMed revealed that 44% of clinical studies in this field have corresponding published results, which was also discussed.

## Introduction

Stroke constitutes the second leading cause of death worldwide. The only effective current treatment method comprises thrombolysis established in the hyperacute period within several hours after onset,^1^ although even following successful preservation of life, various neuronal disorders remain in most cases and the recovery mainly depends on extensive rehabilitation.^2^ In this context, cell therapy that has been developed over the past several decades represents a promising alternative or supplemental strategy; notably, this approach has already reached the translational stage, with therapeutic results in humans having been discussed.^3–10^ However, although the safety of these procedures has so far been confirmed, their efficacy is less certain.^11^ For example, in their recent meta-analysis Nagpal et al.^12^ reported a trend toward improvement of some functional impairment in patients with stroke administered stem cell therapies. Nevertheless, such improvement was not sufficiently demonstrated in controlled studies.^12^

ClinicalTrials.gov^13^ is the world’s largest clinical trial registration database, which is managed by the US National Library of Medicine and provides information regarding the implementation status of more than 310,000 clinical trials from over 200 countries. Additionally, the International Clinical Trials Registry Platform (ICTRP) portal site managed by the World Health Organization (WHO) can be searched for data from 17 primary registries worldwide.^14^ Ideally, all available registries should be investigated if possible; nevertheless, the use of WHO ICTRP in combination with ClinicalTrials.gov provides data from an additional 17 registries around the world, thereby providing broad coverage to enhance the value of a report. These websites provide information regardinzg various attributes, such as target diseases, sponsors, and enrollment of the subjects of planned clinical trials including work in progress. Although these registry data do not provide comprehensive results of clinical trials, this information enables trend analysis of the clinical development of the planned study. From the standpoint of manufacturers and funding in terms of making the maximum use of limited resources, such analysis is extremely important to provide a better understanding of the level of competition, kind of materials used, and degree of testing planned and conducted in practice in clinical trials. We further consider that the suitable analysis of registry registration data constitutes an effective means to obtain timely information because there can be a long delay between study initiation and the reporting of test results. Therefore, in the present report, rather than compiling a review of stroke, we used ClinicalTrials.gov and WHO ICTRP to extract stroke clinical trial data and highlighted various issues regarding clinical trial trends.

## Results

### Study design

We searched ClinicalTrials.gov and selected 77 studies on regenerative medicine for stroke. In addition, we surveyed ICTRP in the same manner and identified 14 additional studies excluding duplications with the ClinicalTrials.gov registered (NCT) studies, thereby obtaining 91 studies in total. We excluded 13 studies involving sickle red blood cells and/or for only young children; the resulting 78 studies were used for analysis of the translational trends of cell therapy for stroke.

### Trend analysis from registered data

**Fig. 1:**
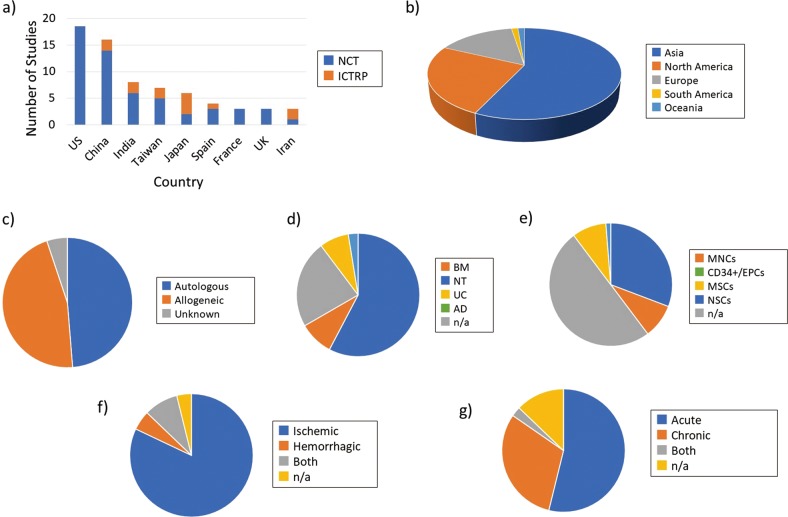
Classification and analysis by various attributes of clinical trials of cell therapy for stroke (I). **a** Comparison of number of clinical trials on stroke by country of origin. The top nine countries are shown in this graph. Blue color-coded bars depict the trials registered in ClinicalTrials.gov. Orange color-corded bars depict the trials supplemented from the ICTPR portal site. **b** Pie-chart analysis of the number of studies by continent. **c** Percentage of cell origin used (autologous or allogeneic) relative to the total number of studies. **d** Percentage of each cell source organ used relative to the total number of studies. **e** Percentage of each cell type used relative to the total number of studies. **f** Percentage of subjects recruited for each disease state (ischemic or hemorrhagic) relative to the total number of studies. **g** Percentage of each stroke condition (acute or chronic) in recruited subjects relative to the total number of studies

Figure 1a shows the analyzed results by country. The United States (US), which manages ClinicalTrials.gov, and China had 18 and 16 studies, respectively, occupying the top rankings. In third place was India, followed by Taiwan and Japan. Asian countries had relatively more study registrations in the ICTRP. Figure 1b shows a pie-chart by continental area. Asia had more than twice as many studies as North America, indicating that this area had a focus on stroke cell therapy.

**Fig. 2:**
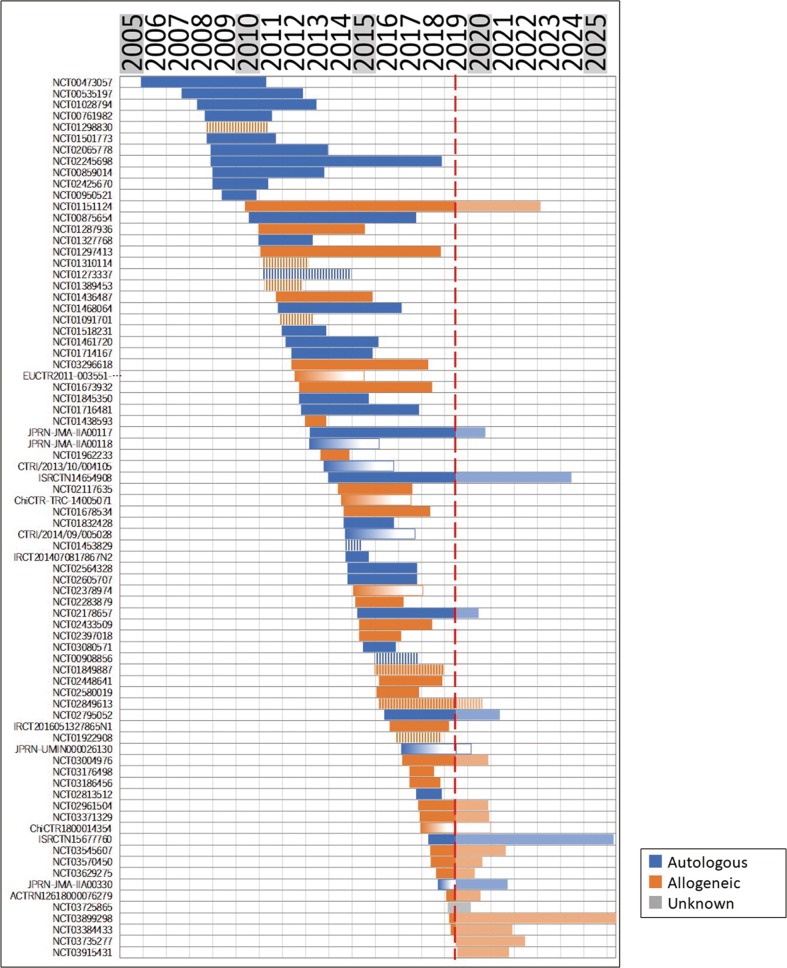
Classification and analysis by various attributes of clinical trials of cell therapy for stroke (II). Each study was color-coded by the corresponding origin of cells and displayed from the start date to the (planned) completion date, sorted by start date in chronological order. Red line: data download date (8 July 2019). In cases where the completion date was not available, a gradated bar over 3 years is shown. Shaded bar on the right of the red line: current year (2019) to 2025. A bar is displayed if the trial was registered until the data download date. Note that we were not able to depict whether the trial continued or was halted prematurely after the data download date. Vertical-striped bar indicates “terminated” or “withdrawn” study

We next classified the entire list of studies by the origin (autologous or allogeneic) of each cell source used (Fig. 1c). The results showed that studies using cells of autologous origin comprised more than half (52%), whereas 44% used allogeneic cells. To further analyze the clinical research trends shown in these studies chronologically, all studies were arranged in order of corresponding starting date and plotted from the start date to the completion date, and color-coded according to the origin of the cell used (Fig. 2). This indicated that until 2009 autologous cells were dominantly used, whereas the number of studies using allogeneic cells increased from 2010. Although some short-term increases and decreases occurred thereafter, no tendency to converge to a single origin type was observed.

Studies were also classified by the cell source organ used (Fig. 1d). We found that 60% of the studies used bone marrow (BM)-based cells, with the umbilical cord (UC) accounting for an additional 22%, followed by adipose tissue (AD) and neural tissue (NT). Alternatively, classification by cell type (Fig. 1e) indicated that mesenchymal stem cells (MSCs) were predominantly used in almost half of the examined studies, followed by mononuclear cells (MNCs) accounting for approximately 30%.

The kind of disease condition targeted by the individual research also represents an important question. Figure 1f shows whether the target stroke was ischemic or hemorrhagic. The overwhelming majority of studies (approximately 80%) targeted ischemic stroke. Subsequently, we analyzed whether the condition was acute or chronic (Fig. 1g). According to the description of inclusion criteria for each study, we classified the stage of each subject as “acute” (within 90 days after onset) or “chronic” (exceeding 90 days). As a result, we found 40 studies for acute phase and 23 studies for chronic phase. We also classified and analyzed the prevalence of a subacute phase in the acute phase.

**Fig. 3:**
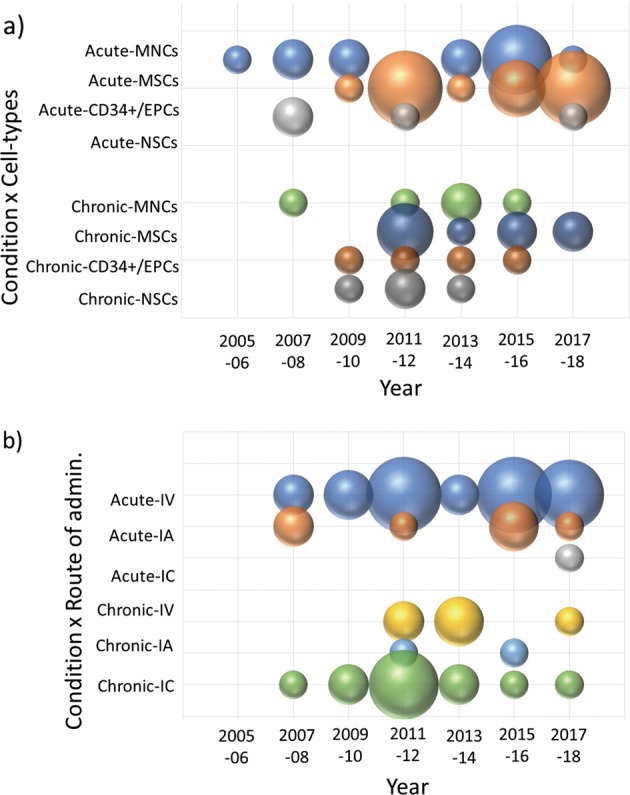
Transition analysis of clinical trials of cell therapy for stroke by cell type used and route of administration. **a**, **b** Bubble chart showing 2-year transitions of the number of studies by cell type **a** or route of administration **b** chosen for treatment of acute or chronic stroke

Figure 3a shows the transition every 2 years regarding the type of cells chosen for application in the acute or chronic phase. In the acute phase, MNCs (including naive cells) were initially considered, whereas MSCs have become mainstream since 2011 and currently remain under examination. The use of CD34+/endothelial progenitor cells (EPCs) and neural stem cells (NSCs) has been minor. In contrast, although MNCs were initially chosen for use in the chronic phase as well, the proportion of studies incorporating MSCs is relatively low compared to that of acute phase studies, indicating that CD34+/EPCs and NSCs were selected instead.

**Fig. 4:**
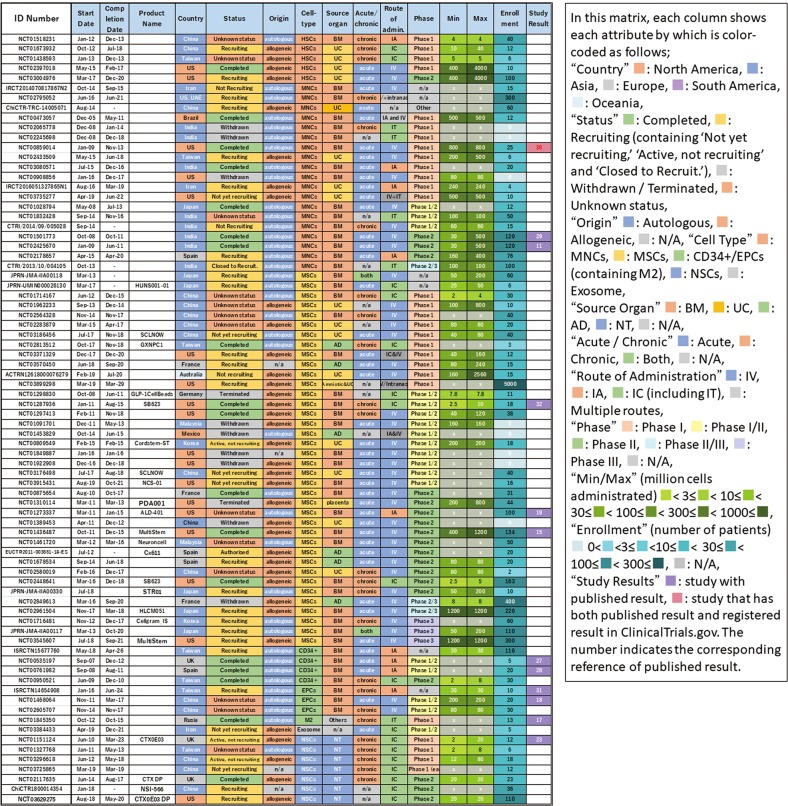
Classification and analysis by various attributes of clinical trials of cell therapy for stroke (III). This matrix indicates each study as a row and each attribute as a column, sorted by cell type

Figure 4 shows the clustering of cell types for matrix analysis of autologous/allogeneic, used cells, acute/chronic, route of administration, and development phase. It can be seen that the combination of acute stroke and intravenous (IV) administration predominates in a large number of studies with MNCs and MSCs, whereas intracranial (IC) administration was mostly used for targeting chronic stroke with NSCs. In addition, studies with BM-derived MNCs and CD34+/EPCs were all autologous. Furthermore, we compared which route of administration was adopted in the acute/chronic phase (Fig. 3b). In the acute phase, most studies adopted IV from the beginning, with less intraarterial (IA) and IC administration. In contrast, in the chronic phase, IC administration was predominantly adopted and has been continuously carried out since 2009 up to the present, although a few studies used IV with rare IA administration. In summary, we found a trend that IV administration was adopted for the acute phase, whereas IC was used for the chronic phase.

**Fig. 5:**
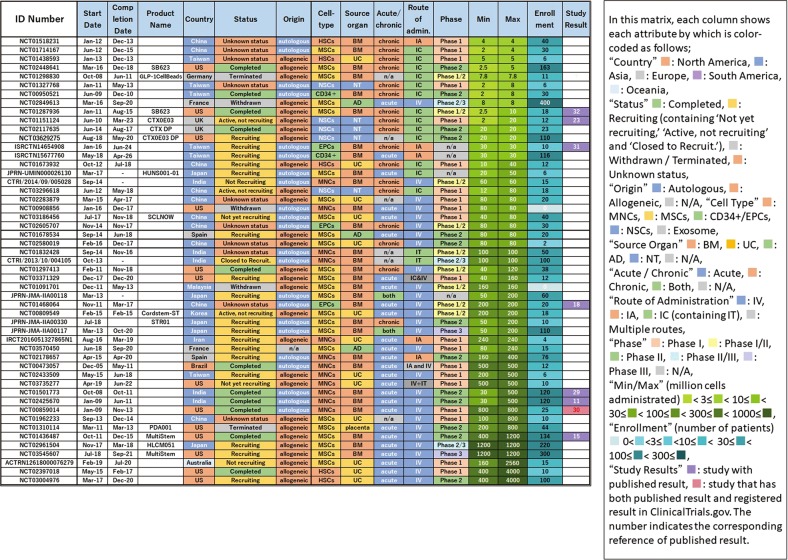
Classification and analysis by various attributes of clinical trials of cell therapy for stroke (IV). This matrix indicates each study as a row and each attribute as a column, sorted by maximum number of cells administrated. This figure shows each study describing the number of administered cells in the study plan

We selected trials in which the number of administered cells was described in the study plan (52/78) and analyzed the number of cells administered. We chose the maximum dose if plural doses were described because we considered that it represented the upper limit considered to be testable in that study. Figure 5 shows the results sorted by maximum number of cells administered. We found a difference of 1000-fold between trials regarding the maximum dose used. The number of cells administered by IV was large, and most cases involved acute administration of MNCs or MSCs. In addition, allogeneic was dominant in cases where the number of cells administered was 500 or more. In comparison, cases with few cells used IC, with the majority applied in chronic disease. In particular, NSC was administered only with IC.

### High clinical staged products

**Fig. 6:**
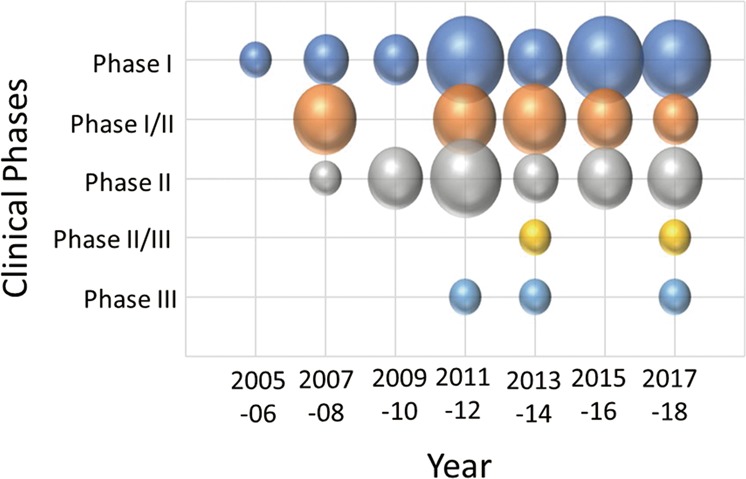
Transition analysis of clinical trials of cell therapy for stroke by translational phase. Bubble chart showing 2-year transitions of the number of studies by translational phase

Stage-up of a clinical trial to phase III suggests that the effectiveness of the product would be expected. We selected 65 studies containing description of clinical phases and analyzed the relationship between the study phase and the start year of the individual studies using a bubble chart (Fig. 6). Phase I, phase I/II, and phase II studies accounted for up to 60 studies in total, with phase I studies starting from 2005, phase I/II from 2007, and phase II from 2008. The higher-stage studies started from 2012, representing only five studies in total, comprising two in phase II/III and three in phase III. The breakdown of these five cases was three autologous and two allogeneic (same product), all originating from BM, and all but one MNC study using MSCs.

**Fig. 7:**
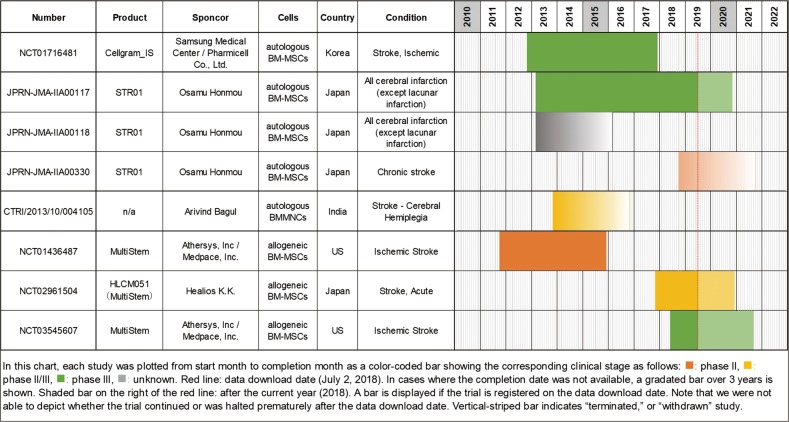
Summary of all five high-clinical trial stage-related studies with their ladder chart. Red line: data download date (8 July 2019). In cases where the completion date was not available, a gradated bar over 3 years is shown. Shaded bar on the right of the red line: after the current year (2019). A bar is displayed if the trial is registered on the data download date. Note that we were not able to depict whether the trial continued or was halted prematurely after the data download date. Vertical-striped bar indicates “terminated” or “withdrawn” study

We extracted all studies associated with these higher-stage studies from all datasets (excluding terminated or withdrawn studies) and summarized them along with their ladder chart (Fig. 7). We obtained three additional studies associated with STR 01 and MultiStem. A phase III study of Cellgram IS derived from autologous BM-MSCs was conducted from 2012 to 2017 against ischemic stroke. Although this study was planned to be completed by the end of 2017, the data had not yet been updated by the time we downloaded, and it has not yet been published to date. A phase III study of STR 01 derived from autogenous BM-MSCs for all cerebral infarction (except lacunar infarction) are planned to be carried out from 2013 to 2020, and the follow-up study are also conducted accordingly. Furthermore, a phase II study for chronic stroke was newly initiated in 2018. In India, a phase II/III noncontrolled study (CTRI/2013/10/004105) was conducted in 2013 in order to examined the effects of autologous BMMNCs administered via intrathecal administration for cerebral hemiplegia. No description of the completion date was provided, and we could not find any further information. A phase II study of MultiStem, which is derived from allogeneic BM-MSCs, was completed in 2015, and the results were published.^15^ Subsequently, narrowing the window between 24 and 36 h after onset as the inclusion criteria compared to the phase II study, a phase III study has just begun in the US in 2018. MultiStem is termed HLCM 051 in Japan, and the phase II/III study was initiated in 2017. Notably, to date, we have been unable to identify a trial where phase III completion has been confirmed. Phase III of Cellgram IS should have already been completed per the schedule, but no report of completion has yet been provided. And, no authorized products have yet been confirmed for clinical cell therapy of stroke.

### Result information obtained from the registry data

The outcomes of clinical trials, especially information regarding safety, are important for numerous reasons yet these results are rarely reported in registries, such as ClinicalTrials.gov and ICTRP. However, in discontinuation studies the reasons may be more likely to be provided. In particular, among the 11 discontinuation studies in this analysis, nine included the reasons. Notably, none were related to safety issues (Supplementary Table 1).

Although study results should be essentially reported to the registry, they are rarely attached. Of the 78 relevant studies no study result was included in ICTRP and only one result report was attached to a study in ClinicalTrials.gov. However, if the clinical trial ID numbers are described in the abstract of the relevant articles, we can search them on PubMed. Thus, we screened them using ID numbers of 78 studies and could identify corresponding publication to 12 clinical trial results. This sample number is not enough for comprehensive analysis, and the evaluation of study outcomes is outside of the main focus of this article. Nevertheless, here we list information regarding safety and severe adverse events from these studies, which are considered particularly important factors, summarized in Supplementary Table 2. Overall, we found that these data were generally consistent with the results of previous studies on the safety of cell therapy for stroke to date.^16^ That is, the reported severe adverse events, including recent data,^17–19^ were not related to the cell therapy or were mainly related to the route of administration. In addition, the cells themselves were reported to be relatively safe; moreover, immunological adverse events for allogeneic cells were not observed in these articles.

### Estimation of the influencing factors from observations over time

As we have previously reported,^20^ a chronological analysis of studies visually represents changes in the number of studies per duration. Figure 2 shows a relatively large gap between 2009 and 2010. A suspected cause is the effects of the STEPS guidelines published in 2009 (ref. ^21^); i.e., we suspected that it might take considerable time to comply with these guidelines. Therefore, we hypothesized that if STEPS had been implemented, improvement might be recognized in the recommended factor. Consequently, as an indicator of the research quality that can be extracted from registry data of the STEPS recommendations, the percentage of studies with controls, and the description of using a vehicle for within-study comparators, we investigated whether the percentage of equivalent rehabilitation descriptions in both test groups increased following the 2010 gap. However, we found that almost no change in the descriptions had occurred since 2010 compared to the previous ones in 2009 (Supplementary Fig. 1).

## Discussion

The purpose of the present analysis was not to outline the safety and efficacy in the reviewed trials but rather to determine the tendencies of attributes in the planned clinical trials. In our previous work,^20^ we analyzed ClinicalTrials.gov as the only information source. However, because ClinicalTrials.gov is a US database, there is some concern that domestic registration bias may exist; i.e., that comprehensiveness of trials other than those of the home country may decline. Accordingly, in the present study, we obtained the data from ClinicalTrials.gov along with the WHO ICTRP portal site as information sources and analyzed these collectively. The use of WHO ICTRP in combination with ClinicalTrials.gov can be expected to improve the coverage close to the ideal because the former receives data from 17 major registries around the world including ClinicalTrials.gov.

For the current study, we searched WHO data under the same conditions for the purpose of improving coverage and found 14 additional studies (19%), as shown in Fig. 1a. These additional studies were mostly registered from Asian countries such as China, Taiwan, India, and Japan (12/14). Consequently, Asian countries were dominant (56%) in our overall study set compared with North America and Europe (24% and 18%, respectively). Notably, Feigin et al.^22^ reported that the disability-adjusted life year (DALY) of patients with stroke is greater in East Asia than that in the United States and Western Europe combined. It is reasonable that clinical trials are actively carried out in East Asian countries, where economic activity is sound, and the importance of stroke is relatively high in terms of burden of disease. By using the WHO registry (ICTRP) portal site in combination with ClinicalTrials.gov, we believe that coverage in clinical areas with relatively small involvement in the US, such as stroke, can be complementarily improved.

The use of autologous cells is advantageous, owing to the lack of concern for immune rejection and strict quality control, but often exhibits quantitative limitations and requires a relatively long term for preparation. In comparison, allogeneic cells may be used off-the-shelf and are advantageous for quantitative preparation, but the problem of immune rejection may remain and strict quality control may be necessary for further production. This choice will ultimately depend on the purpose for which the cells are used. Our chronological analysis indicated that studies initially utilized autologous cells, whereas allogeneic cells appeared to be considered after the rudimentary results were obtained. Although respective increases and decreases occurred thereafter, we did not observe any tendency to converge to either the use of autologous or allogeneic cells. Rather, both cells were shown to be studied to the same extent, suggesting that the field may not have yet reached a stage of conclusion.

As a result of clustering by cell type in Fig. 4, we identified many studies using MNCs and MSCs, which were popularly used for acute phase and mainly administered via IV. This may derive from the fact that BM, UC, and AD, the origin of MNCs/MSCs, are easily available as cell sources. These tend to be used in large quantities in IV. Both MNCs and MSCs exhibit properties of angiogenesis, anti-inflammatory/immune regulation, and trophic effects.^23^ It is thought that CD34+ cells were first attempted in that context, which were then replaced by EPCs. These were basically autologous cells except those of UC origin, which were allogeneic. After 2010, MSCs were favored rather than MNCs, increasing the use of allogeneic cells. Although MSCs show more immunomodulatory properties, MNCs were preferred because they are easier to prepare and expected to be effective as hematopoietic stem cells in stroke. The reason why these cells are used in the acute phase as IV is considered to be that the main mechanism of action is to suppress the inflammatory response by systemic immune regulation. In this regard, it is expected that some currently ongoing phase III or phase II/III studies using MSCs against acute stage stroke (e.g., Cellgram IS, STR 01, and MultiStem in Fig. 7) may provide answers regarding their efficacy.

Although NSCs are expected to promote nerve repair around the damaged region, only five studies using NSCs were identified. Among these, the safety of CTX-DP was investigated in the first-in-man study (NCT 01151124) and the results were reported in 2016 by Kalladka et al.^24^ In addition, a phase II study for CTX-DP (NCT 02117635) was launched in 2014 and should be completed in 2017, although the results have not yet been published. As the inclusion criteria of this phase II study included a subject with stroke at 2–3 months after onset, we classified this study as representing acute stage instead of chronic phase according to our classification in the present study. Finally, NSI-566 is a human fetal spinal cord-derived cell line developed in China. Its phase I trial (NCT 03296618) was launched in 2012 and completed last year; phase II has been implemented in China (ChiCTR 1800014354). For NSCs, systemic immune regulation and neuroprotection are expected in the acute phase, with neural repair promotion and apoptosis suppression being expected in the subacute phase. Conversely, in the chronic phase there is no involvement of systemic immunity; thus, the intended outcomes of cell therapy include neurogenesis, synapse formation, and plasticity enhancement.

Studies using MSC in the chronic phase have also been examined but no clear trend was observed. Although only SB623, representing modified MSCs, has previously shown slightly efficacious results,^25^ it has recently been reported that a phase II study using SB623 could not afford significant efficacy.^26^ In addition, a phase III study using STR 01 is underway for all cerebral infarctions (except lacunar infarction), whereas a phase II study was started separately for the chronic phase (JPRN-JMA-IIA00330). The efficacy of STR 01 for the chronic phase thus cannot yet be ascertained.

**Fig. 8:**
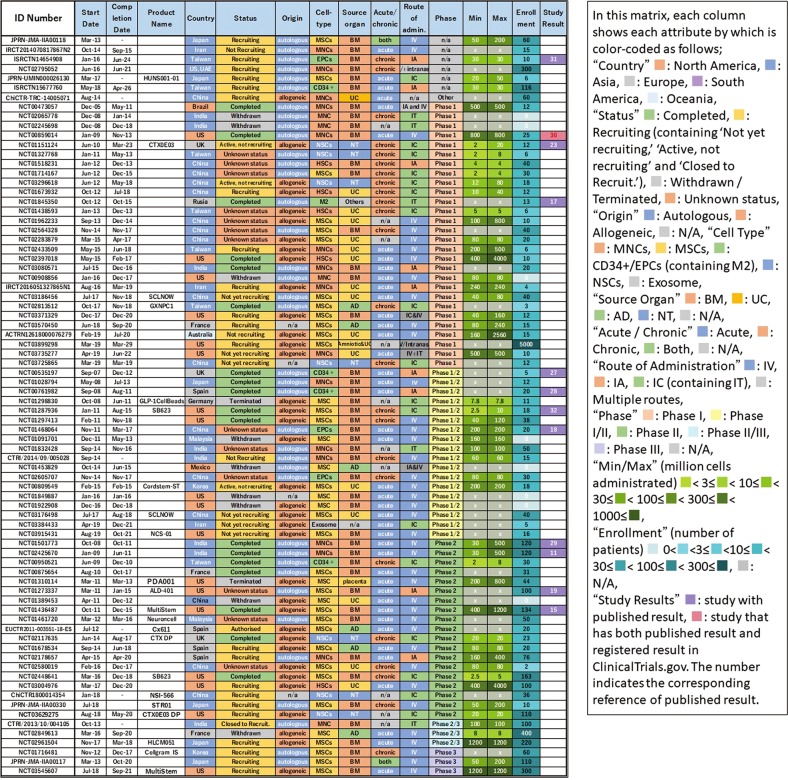
Classification and analysis by various attributes of clinical trials of cell therapy for stroke (V). This matrix indicates each study as a row and each attribute as a column, sorted by translational phase

Overall, the number of clinical studies for cell therapy in the field of stroke is small and no products are yet approved by the authorities for this purpose. For example, in the cartilage repair arena analyzed in our previous study,^20^ we identified 14 large, randomized, and controlled phase III studies with enrollments of over 100 patients. In contrast, for stroke, only two controlled Phase III studies with over 100 enrolled patients are registered and none of them has been completed (Fig. 8). This indicates that a major problem in the stroke field is the lack of a large-scale randomized controlled clinical trial. This may be due to an insufficient understanding of the natural history of stroke and/or the heterogeneity of the disease condition including certain comorbidity among the patient population. Thus, it can be considered that cell therapy against stroke represents a premature area compared with cartilage repair.

In addition, we identified 12 results report documents corresponding to it out of a total of 78 cases by tracing the ID number of the clinical trial registry. Some of these documents contain the obtained short-term results on clinical trials and the long-term follow-ups, which will be completed in the future. Therefore, the results vary depending on how the parameter is measured. The report rate is 44% if only the completed reports are counted (8 reported of 18 total studies). This percentage is consistent with previous reports that the trial report publication rate is 20–50%.^27^ Although various analyses are possible, here, using stratification analysis by phase, the results show that Phase 1/2 and Phase 2 are 60% and 43%, respectively, whereas Phase 1 is low at 33%. Because Phase 1 aims to test safety, in addition to the difficulty in reporting negative results, a lack of novel topics when trials are performed use similar cell materials also suggests that reporting in such cases may be difficult. If such is the case, however, based on the recognition that the test results contribute to our knowledge regarding human response, we believe that these should be disclosed in the registry.

Overall, the analysis of reports using clinical trial registry information allowed trends to be highlighted. Moreover, even though few actual results are registered in the clinical trial registry, tracking the publication of each trial lead to the identification of several results reports. Normally, 100% of test results should be reported within a certain number of years at the end of each trial although in reality this is seldom the case. We believe that a research focused on result reporting for clinical trials conducting research on regenerative medicine is necessary.

Recently, a phase 1 plan was registered in ClinicalTrials.gov comprising over 1000 patients and involving the administration of human-derived tissue for a wide range of diseases. However, no specific details were elaborated, such as disease kind or stage on which treatment would be based or on which kind of symptoms the patients are recruited. Such plans are not realistic and there is an element of advertising that appears to deliberately exploit the publicity of ClinicalTrials.gov. This approach, diverging from the original purpose, lessens the value of the registry and may also cause the general public to distrust regenerative medicine itself. Rather, the issues discussed above regarding the inclusion of relevant, comprehensive, and timely information should be taken into consideration when using the registry for clinical trial registration.

In summary, we extracted 78 types of attribute data from the clinical trial registry of cell therapy for stroke and comprehensively analyzed these, revealing that MNCs/MSCs are used in relatively large volumes via IV in the acute phase, whereas in the chronic phase, NSCs are also used albeit generally through IC administration of relatively small doses. It is expected that the process of trial and error will continue in the area of regenerative medicine with regard to stroke research, as it appears not to have fully matured compared with other areas of study, as evidenced by the small number of well-controlled large-scale phase III trials. Thus, further tracking of research trends in this area appears to be warranted in the future.

## Methods

We searched the entire database at ClinicalTrials.gov on 8 July 2019 using the following search terms: condition = stroke; other = “stem cell” OR “regenerative” OR “cell therapy”. Among the identified and downloaded 272 studies as CSV format, we excluded studies using only surgical procedures, low molecule weight drugs, protein drugs, or scaffolds by carefully reading the descriptions of the individual studies, and selected 74 studies corresponding to cell therapy, which administered cells to humans to examine their safety and efficacy. Furthermore, the relevant studies were re-surveyed using the product name, development code, and/or sponsor’s name described in the 74 studies as search terms and were selected manually. Three additional studies were found to be incorporated into the 74 studies.

Furthermore, we surveyed the ICTRP portal site on 8 July 2019, and the relevant studies were selected in the same manner and downloaded as XML format. After carefully excluding duplication with those obtained from ClinicalTrials.gov, 14 additional studies were chosen. From the total of these 91 subjects, excluding those 13 studies targeting sickle blood cells and children, 78 studies were selected for subsequent analysis.

We performed the processing of datasets using Microsoft Excel 2016 manually as follows; we recorded the organ used as the cell source, cell type, product name (if any), and country where the clinical study was performed. The cell sources used were classified as BM, NT, AD, UC, and others. BM included peripheral blood and fractionated blood cells. AD included all materials described as adipose and fat. UC set contained stem cells derived from UC blood, placenta, and amniotic membrane/fluid. All cell sources classified as UC were regarded as “allogeneic”.

The cell types used were classified as MNCs, MSCs, CD34+/EPCs, NSCs, and others according to the description. MNCs in our analysis included naive cells. BM stem cells that could be identified as MNCs by their description were classified as MNCs and the others were classified as MSCs.

According to the description of each inclusion criteria, a subject within 90 days after onset was classified as acute phase, and those exceeding it were classified as chronic phase. According to the description of each study, it was classified as IV, IA, or IC (including intrathecal).

For examining the transition analysis of study design (controlled and noncontrolled study), we used a total of 67 studies excluding withdrawal or terminated studies.

### Reporting Summary

Further information on research design is available in the Nature Research Reporting Summary linked to this article.

## Supplementary information

Supplementary Table and Figures

Reporting Summary

## Data Availability

The data is available publically through the WHO ICTRP portal and ClinicalTrials.gov. The raw data that support the findings of this study are available from the corresponding author upon reasonable request.

## Code Availability

We performed the generation and processing of datasets described above using Microsoft Excel 2016, without any special coding.
