# Supplementary material for: Trends in clinical trials for stroke by cell therapy: data mining ClinicalTrials.gov and the ICTRP portal site
Source: NPJ Regen Med. 2019 Nov 6;4:20. doi: 10.1038/s41536-019-0082-7 (PMC6834621; doi:10.1038/s41536-019-0082-7)
Supplement: Supplementary file 1 — Supplementary Table and Figures [file 41536_2019_82_MOESM1_ESM.pdf]

Supplementary table 1

Summary of reasons on terminated or withdrawn 11 studies found in ClinicalTrials.gov. or ICTRP

NCT Number	Recruitment	Location	Acute or Chr	hemorrhagic	Sponsor/Collaborators	Funded Bys	Remarks
**NCT01298830**	Terminated	Germany	hemorrhage	hemorrhage	CellMed AG, a subsidiary of BTG plc.	Industry	Need for improvement of study medication. Safety data collected sufficient. No further gain in knowledge expected.
**NCT02065778**	Withdrawn	India	chronic	-	Neurogen Brain and Spine Institute	Other	n/a
**NCT02245698**	Withdrawn	India	-	-	Neurogen Brain and Spine Institute	Other	n/a
**NCT01310114**	Terminated	US	acute	Ischemic	Celularity Incorporated|Celgene Corporation	Industry	Study terminated by sponsor
**NCT01389453**	Withdrawn	China	acute	cerebral infa	General Hospital of Chinese Armed Police Forces	Other	There were not enough number of patients recruited till DEC 31th in 2012.
**NCT01091701**	Withdrawn	Malaysia	acute	ischemic	Stempeutics Research Pvt Ltd|Stempeutics Research Malaysia SDN BHD	Industry|Oth	Business reasons
**NCT01453829**	Withdrawn	Mexico		ischemic str	Ageless Regenerative Institute|Instituto de Medicina Regenerativa, S.A. de C.V.	Industry	no enrolled participants
**NCT00908856**	Withdrawn	US	acute	ischemic	University of California, Irvine|University of Ca	Other	Not funded
**NCT01849887**	Withdrawn	US	acute	Ischemic	University of California, Irvine	Other	Study was never started
**NCT02849613**	Withdrawn	France	acute	ischemic	University Hospital, Grenoble|European Commission H2020 program|University	Other	Not authorized by Regulatory agencies - new protocol phase 1a in progress
**NCT01922908**	Withdrawn	US	acute	Ischemic	Sean Savitz|The University of Texas Health S	Other	Plan to change the trial design

Supplementary table 2

Summary of safety on 12 studies linked to the articles with the corresponding results in ClinicalTrials.gov. or ICTRP

NCT Number	Cells	Location	Summary of Safety & SAE	Conditions	Route	Phases	Enrollment	Start Date	Ref.
NCT00535197	autologous CD34+ subset bone marrow stem cell	UK	No patient died.<br>SAE: renal dysfunction / pneumonia (not related to the intervention)	Stroke, Acute / Infarction, MCA	IA	Phase I/II	5	Sep-07	①
NCT00761982	autologous bone marrow stem cells (CD34)	Spain	No death, stroke recurrence, or tumor formation.<br>SAE: an isolated partial seizure (2)	Stroke, Acute / Infarction, MCA	IA	Phase I/II	20	Sep-08	②
NCT01501773	autologous bone marrow mononuclear cells	India	No SAE was observed during the study.<br>a small new infarct after 4-6 wk (clinically silent)<br>no evidence of tumour formation (PET scan at one year)	Acute Stroke	IV	Phase II	120	Oct-08	③
NCT00859014	autologous bone marrow mononuclear cells	US	There were no significant hemodynamic or pulmonary changes during the infusion.<br>There were no seizures and no evidence of new cerebral infarcts.<br>a pulmonary embolism (PE) related to the stroke (not	Ischemic Stroke	IV	Phase I	25	Jan-09	④
NCT02425670	autologous bone marrow mononuclear cells	India	Kaplan-Meier survival curve, the AE and SAE were comparable between the 2 arms.<br>epileptiform discharges (3 in BMSCs arm)	MCA Infarction / Anterior Cerebral Artery Infarction	IV	Phase II	120	Jan-09	11
NCT01151124	human neural stem cells (allogeneic)	UK	SAE related to the neurosurgical procedure: Extradural haematoma (1; asyptomatic), Subdural haematoma (1; asyptomatic), Minor bleed at the burr hole on MRI (2)<br>All other SAE were related to incidental or known medical conditions.<br>No event was judged to be attributable to CTX-DP.<br>All patients who received treatment were HLA negative before and after intervention.	Stroke	IC	Phase I	12	Jun-10	24
NCT01287936	modified stem cell (allogeneic bone marrow-derived)	US	All patients in the safety population experienced at least 1TEAE.<br>Six patients experienced 6 serious TEAE; 2 (Headache) were probably or definitely related to surgical procedure; none were related to cell treatment.<br>There were no dose-limiting toxicities or deaths.<br>During the study, no patient developed antibodies to SB623 HLAs.	Chronic Ischemic Stroke	IC	Phase I/II	18	Jan-11	⑤
NCT01273337	autologous ALDHbr cells (bone marrow-derived)	US	SAE: Convulsion, Cerebral hemorrhage, Cerebrovascular accident, Hemorrhagic transformation stroke, Syncope, Deep vein thrombosis, Hypertension, Hypotension, Angina unstable, Sick sinus syndrome, Tachycardia, Ventricular tachycardia, Pulmonary embolism, Dyspnea, Pneumonia, Urinary tract infection, Thrombocytopenia, Retinal artery embolism, Chest pain, Astrocytoma, low grade, Anxiety, Craniectomy<br>No infusional or allergic reactions and no difference in treatment emergent adverse events.	Stroke|Ischemic Stroke|Stroke in Middle Cerebral Artery (MCA)	IC	Phase II	100	Mar-11	19
NCT01436487	BM-adherent progenitor cells (allogeneic bone marrow-derived)	US	Life-threatening adverse events, death, and secondary infections were not significantly different between arms.<br>There were no infusion related allergic reactions and no neurological worsening in either group.<br>Mortality was not different between the arms.	Ischemic Stroke	IV	Phase II	134	Oct-11	15
NCT01468064	autologous bone marrow mesenchymal cells/endothelial progenitor cells	China	No treatment - emergent adverse events relating to bone marrow aspiration or cell transplantation were found in any of the three groups.<br>No event was judged to be attributable to stem cell transplantation.	Stroke / Infarction, MCA	IV	Phase I/II	20	Nov-11	18
NCT01845350	autologous M2 macrophage	Russia	No serious short-term AEs during the first 3 days following cell administration.<br>No cases of allergic reactions, local hematoma, or infections at the site of the lumbar puncture; meningeal reactions; systemic inflammatory complications; or complications related to toxicity were noted after the injection.<br>No cases of neurological worsening, new/recurrent ischemic or hemorrhagic stroke, or seizures.	Ischemic Stroke / Hemorrhagic Stroke	IT	Phase I	13	Oct-12	17
ISRCTN14654908	autologous circulatory derived CD34+ cells	Taiwan	procedural safety was 100% with all patients uneventfully discharged	old ischemic stroke	IA	Phase I	30	Jan-16	⑥

Supplementary references

① Banerjee, S. *et al.* Intra-arterial immunoselected CD34+ stem cells for acute ischemic stroke. *Stem Cells Transl Med*. **3**, 1322-30 (2014).

② Moniche, F. *et al.* Intra-arterial bone marrow mononuclear cells in ischemic stroke: a pilot clinical trial. *Stroke.* **43** 2242-4 (2012).

③ Prasad, K. *et al.* Autologous intravenous bone marrow mononuclear cell therapy for patients with subacute ischaemic stroke: a pilot study. *Indian J Med Res.* **136** 221-8 (2012).

④ Savitz, S.I. *et al.* Intravenous autologous bone marrow mononuclear cells for ischemic stroke. *Ann Neurol.* **70** 59-69. (2011)

⑤ Steinberg, G. K. *et al.* Two-year safety and clinical outcomes in chronic ischemic stroke patients after implantation of modified bone marrow-derived mesenchymal stem cells (SB623): a phase 1/2a study. *J. Neurosurg.* **2018**, 1-11, (2018).

⑥ Sung, P. H. *et al.* Intra-carotid arterial transfusion of autologous circulatory derived CD34+ cells for old ischemic stroke patients - a phase I clinical trial to evaluate safety and tolerability. *Am J Transl Res* **10**, 2975-2989 (2018).

S-fig 1a)

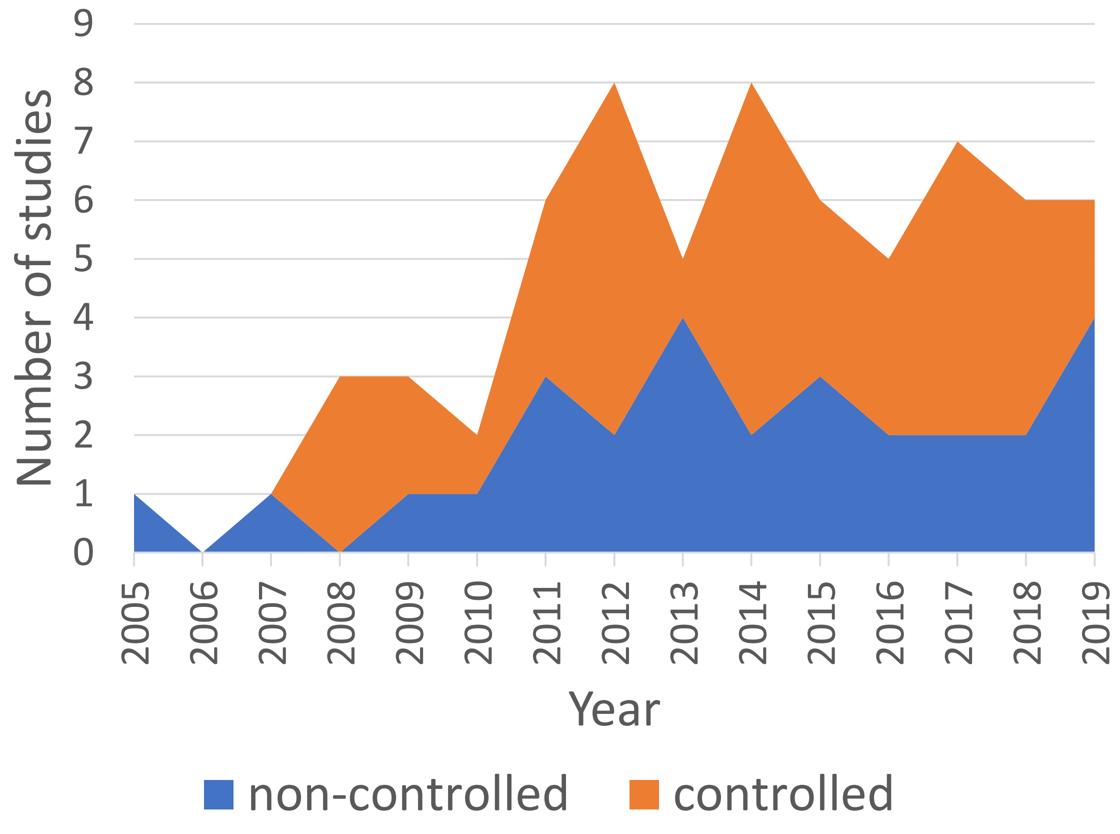

b)

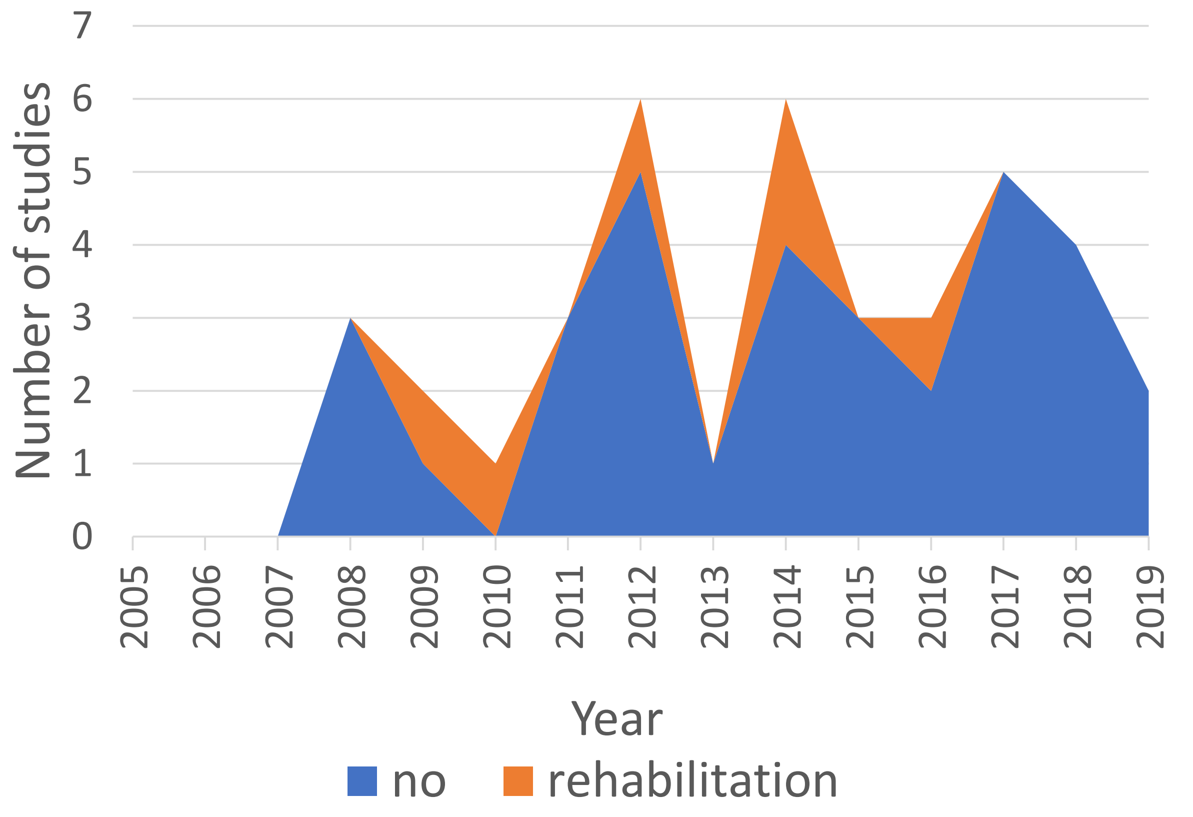

c)

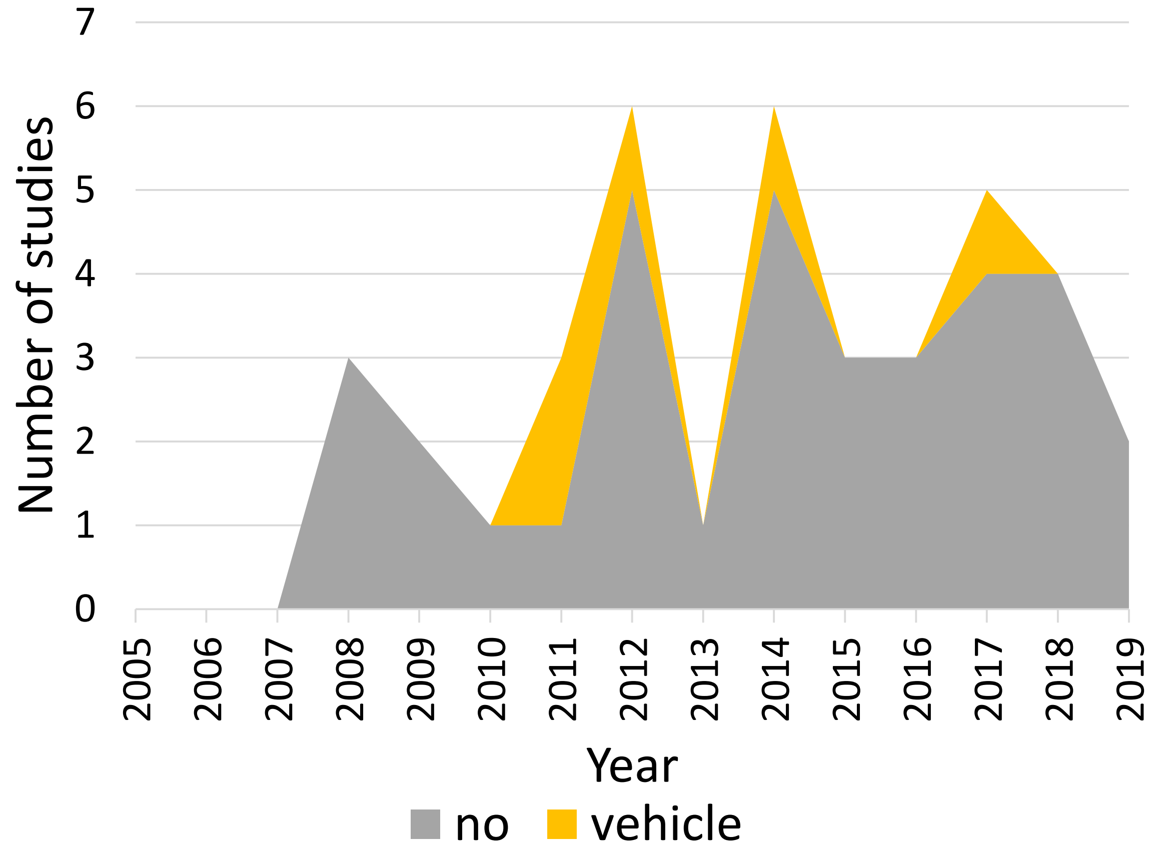

Supplementary Fig. 1 Transition analysis of clinical trials of cell therapy for stroke by study design (controlled or non-controlled study). a Transition analysis of controlled or non-controlled studies. **b** Transition analysis of controlled studies having a description of an equivalent rehabilitation for both study arms. **c** Transition analysis of controlled studies having a description of the use of a vehicle as a comparator.
